# Activation of autophagy flux by metformin downregulates cellular FLICE–like inhibitory protein and enhances TRAIL- induced apoptosis

**DOI:** 10.18632/oncotarget.8048

**Published:** 2016-03-14

**Authors:** Uddin MD Nazim, Ji-Hong Moon, Ju-Hee Lee, You-Jin Lee, Jae-Won Seol, Seong-Kug Eo, John-Hwa Lee, Sang-Youel Park

**Affiliations:** ^1^ Biosafety Research Institute, College of Veterinary Medicine, Chonbuk National University, Iksan, Jeonbuk 54596, South Korea

**Keywords:** metformin, autophagy flux, TRAIL, cancer

## Abstract

Tumor necrosis factor (TNF)-related apoptosis-inducing ligand (TRAIL) is a member of the TNF superfamily. TRAIL is regarded as one of the most promising anticancer agents, because it can destruct cancer cells without showing any toxicity to normal cells. Metformin is an anti-diabetic drug with anticancer activity by inhibiting tumor cell proliferation. In this study, we demonstrated that metformin could induce TRAIL-mediated apoptotic cell death in TRAIL-resistant human lung adenocarcinoma A549 cells. Pretreatment of metformindownregulation of c-FLIP and markedly enhanced TRAIL-induced tumor cell death by dose-dependent manner. Treatment with metformin resulted in slight increase in the accumulation of microtubule-associated protein light chain LC3-II and significantly decreased the p62 protein levels by dose-dependent manner indicated that metformin induced autophagy flux activation in the lung cancer cells. Inhibition of autophagy flux using a specific inhibitor and genetically modified ATG5 siRNA blocked the metformin-mediated enhancing effect of TRAIL. These data demonstrated that downregulation of c-FLIP by metformin enhanced TRAIL-induced tumor cell death via activating autophagy flux in TRAIL-resistant lung cancer cells and also suggest that metformin may be a successful combination therapeutic strategy with TRAIL in TRAIL-resistant cancer cells including lung adenocarcinoma cells.

## INTRODUCTION

Lung adenocarcinoma, one of the most common malignancies among men and women in the world, is the leading cause of cancer related death. Radiotherapy, surgery, and chemotherapy are recognized as front-line channels for cancer treatment. Advanced tumors are occasionally resistant to single agent therapy. Therefore, combination therapy drugs are more effective in curing different types of cancers at advanced stages. Combination chemotherapy can be potent for people with advanced cancers compared to radiation therapy or surgical treatment. The rationale for combination therapy drugs is that these drugs have different mechanisms. Effective and favorable drug combinations have been explored to search for effective combination therapeutics to significantly improve their outcomes in cancer treatment.

Tumor necrosis factor (TNF)-related apoptosis-inducing ligand (TRAIL) is a member of the TNF superfamily. It has drawn extensive attention as an interesting molecule for cancer therapy because it can selectively induce apoptosis of transformed or malignant cells [1, 2]. Tumor necrosis factor-related apoptosis-inducing ligand (TRAIL) has been investigated as one of the most promising anticancer candidates in cancer research because it preferentially induces apoptosis in cancer cells but not in normal cells [3, 4]. Five different receptors have been identified for TRAIL. Of these receptors, only DR4 (TRAIL-R1) and DR5 (TRAILR2) have cytoplasmic death domains engaging in the extrinsic apoptotic pathway upon TRAIL binding [5]. Binding of TRAIL to the death receptor will activate death receptor to recruit Fas-associated death domain protein (FADD) and ultimately procaspase-8 to form death-inducing signaling complex (DISC), leading to the activation of caspases (caspase-8, -9, -10, and -3) [6, 7]. This pathway is conversely governed by the cellular FADD-like interleukin-1b-converting enzyme (FLICE) inhibitory protein (c-FLIP), which harbors structural correlation to procaspase-8 and blocks activation of caspase-8 at the level of DISC formation [8]. Recent evidence has suggested that many types of tumor cells, including human lung adenocarcinoma A549 cells can develop resistance to apoptotic effect of TRAIL [9].

Metformin (1,1-dimethylbiguanide hydrochloride), a biguanide derivative, is the most prescribed drug in the 1950s for the treatment of type 2 diabetes worldwide [10]. Metformin has the ability to suppress hepatic glucose production and trigger glucose uptake in skeletal muscle [11]. In addition, metformin can inhibit the growth of various cancer cell types, including those of prostate [12], breast [13], colon [14], and glial [15] cancer. The direct anticancer effect of metformin is by activating AMPK, a critical cellular energy sensor and suppression of mTOR signaling, leading to the inhibition of protein synthesis and cell proliferation [16].

Apoptosis or type I programmed cell death is morphologically characterized by cell shrinkage, chromatin condensation, DNA fragmentation, and formation of distinct apoptotic bodies [17]. Apoptosis is directed by the following two major pathways: 1) the cell death receptor-mediated (extrinsic) apoptotic pathway, and 2) the mitochondrial-mediated (intrinsic) apoptotic pathway. Both pathways involve the activation of caspase [18]. In cancer cells, activation of apoptotic pathway plays a major protective role against the progression of cancer.

Autophagy is known as type II programmed cell death through lysosomal-dependent degradation mechanism. Its function is to maintain cellular homeostasis by recycling long-live unneeded proteins, eliminating defective cellular components, and sustaining cell survival during long periods of starvation and other stressors [19, 20]. During autophagy, double membrane vesicles (autophagosome) will be developed and consequently fused with lysosome to form auto-lysosome for the degradation and the recycling of cargo [21, 22]. The formation of autophagosome is mediated by coordinated action of Atg12-Atg5-Atg16 complex and microtubule associated protein light chain 3 (LC3-I). LC3-I is conjugated to phosphatidyl ethanolamine to form LC3-II, a widely used autophagy marker [23, 24] and complete autophagosome marker [25–27]. P62/SQSTM1 is an ubiquitin-binding protein. It is also known as an autophagy substrate. P62 is incorporated into autophagosomes via correlating with LC3. It is efficiently degraded in the process of autophagy. Inhibiting autophagy is associated with rapidly increased cellular levels of p62, while decreased p62 levels will result in the activation of autophagy [27]. Antimalarial drug chloroquine (CQ) is also known as an inhibitor of autophagy. It can prevent lysosome acidification and result in the fusion of autophagosome with lysosome, thereby inhibiting late stage of autophagy and degradation of metabolic stress products and inducing apoptosis [28–31].

Although the anti-cancer effect of metformin and the synergistic effect of metformin combined with TRAIL are well known, the molecular mechanisms involved in such effects are currently unclear. Therefore, the objective of this study was to investigate the molecular mechanisms underlying the anti-cancer effect of metformin and the synergistic effect of metformin combined with TRAIL in human lung adenocarcinoma A549 cells.

## RESULTS

### Metformin enhances TRAIL-induced apoptosis in lung adenocarcinoma cells

**Figure 1:**
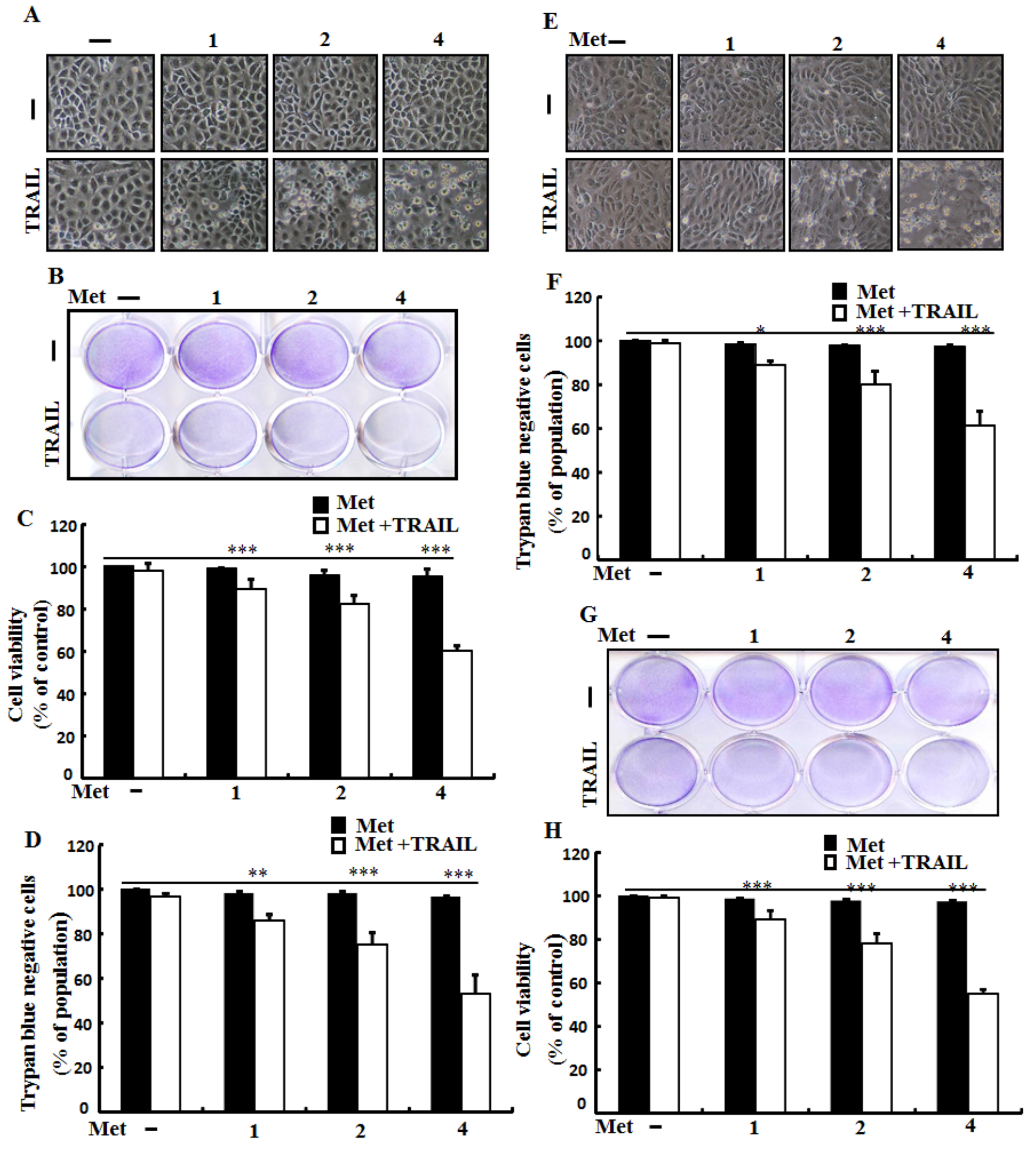
Metformin enhances TRAIL-induced apoptosis in lung adenocarcinoma cells A549, Calu-3 and HCC-15cells were pretreated with metformin at different concentrations (0, 1, 2, and 4 mM) for 12 h followed by treatment with 200 ng/ml of TRAIL protein for an additional 2 h. **A.** and **E.** Cell morphology photographed under light microscope in A549 and Calu-3 Cells (×100); **B.** and **G.** Cell viability determined with crystal violet assay in A549 and HCC-15 Cells; **C.** and **H.** Bar graph showing the average density of crystal violet dye in A549 andHCC-15 Cells; **D.** and **F.** Cell viability determined with trypan blue dye exclusion assays in A549 and Calu-3 Cells. * *p* <0.05 ** *p*<0.01, *** *p* < 0.001: significant differences between control and treatment group; Met: metformin; TRAIL: Tumor necrosis factor (TNF)-related apoptosis-inducing ligand.

To determine the effect of metformin on TRAIL-mediated apoptosis, A549, Calu-3 and HCC-15 lung cells were pretreated with different concentrations of metformin for 12 h followed by treatment with TRAIL protein for an additional 2 h. Cells were photographed under a light microscope to determine the morphological changes. As shown in Figure 1, treatment with metformin or TRAIL alone failed to influence cell viability or only slightly influenced cell viability. After treatment with TRAIL alone, no morphological change was observed compared to the control, indicating that A549, Calu-3, HCC-15 cells were highly resistant to TRAIL. However, co-treatment of TRAIL with various concentrations of metformin significantly reduced cell viability compared to metformin or TRAIL alone. Cell morphology results also revealed enhanced effect of metformin. The combination of TRAIL and metformin enhanced the number of apoptotic cell deaths compared to treatment with metformin or TRAIL alone (Figure 1A). Combined treatment of TRAIL and metformin decreased cell viability and significantly increased apoptotic cell death of A549 cells (Figure 1B, 1C, and 1D). Combined treatment of TRAIL and metformin also decreased cell viability and significantly increased apoptotic cell death of Calu-3, HCC-15 cells (Figure 1E, 1F, 1G and 1H). These results indicated that metformin significantly increased TRAIL-induced apoptotic cell death in TRAIL-resistant human lung A549, Calu-3 and HCC-15 cells.

### Metformin induces autophagy flux and enhanced apoptosis mediated by TRAIL

**Figure 2:**
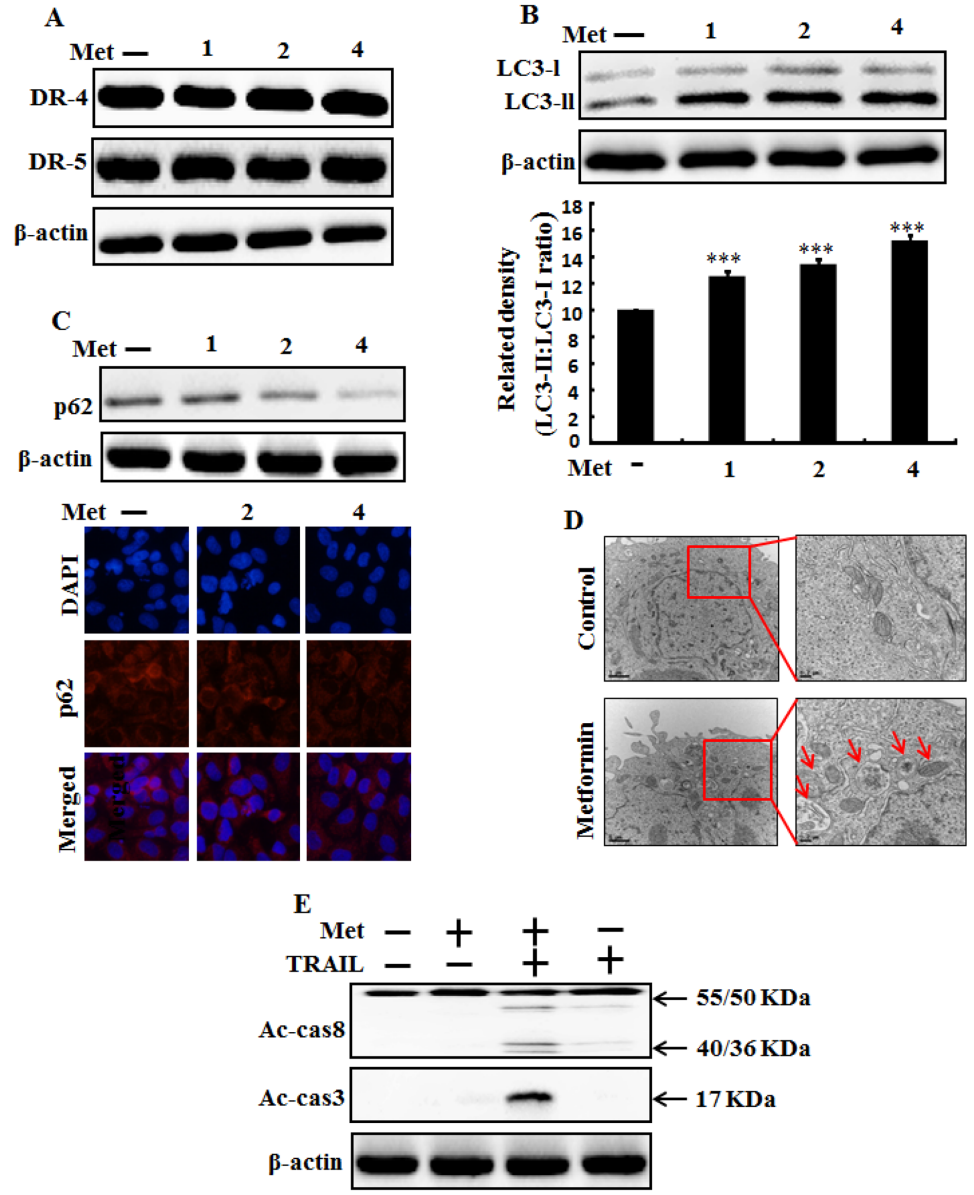
Metformin induces autophagy flux and enhanced apoptosis mediated by TRAIL A549 adenocarcinoma cells were pretreated with metformin at different concentrations (0, 1, 2, and 4 mM) for 12 h. **A.** and **B.** Cells were harvested and analyzed by Western blotting to determine the expression levels of DR-4, DR-5, LC3-II; **C.** Western blot and Representative immunocytochemistry of A549 cells after treatment with metformin for 12 h to determine p62 protein levels; **D.** TEM shows the ultrastructure of cells treated with 4 mM metformin for 12 h. Arrows indicate autophagosomes, including residual digested material and empty vacuoles; **E.** Ac-cas3and Ac-cas8 expression levels determined by western blot analysis. A549 cells were pre-treated with metformin for 12 h and then exposed to 200 ng/ml TRAIL for an additional 1 h. β-actin was used as loading control. *** *p* < 0.001: significant differences between control and treatment group; Met: Metformin; Ac-cas3: Activated caspase 3; Ac-cas8: Activated caspase 8; TRAIL: Tumor necrosis factor (TNF)-related apoptosis-inducing ligand.

To determine the effect of metformin on autophagy flux, lung adenocarcinoma A549 cells were pretreated with different concentrations of metformin for 12 h followed by treatment with TRAIL protein for an additional 1 hr. Whole cell lysates were subjected to Western blot analysis. As shown in Figure 2A, the protein expression levels of TRAIL receptors such as DR4 and DR5 were not changed by metformin at different concentrations. However, the conversion rate of LC3-I to LC3-II was increased by metformin in a dose dependent manner (Figure 2B). Western blot and Immunocytochemistry (ICC) results also showed that various concentrations of metformin decreased the protein levels of p62 (Figure 2C). A TEM assay confirmed that numerous autophagic vacuoles and empty vacuoles were present in the A549 cells treated with 4mM metformin (Figure 2D). The combined treatment of TRAIL and metformin enhanced the expression levels of Ac-cas3 and Ac-cas8 compare to the single treatment with metformin or TRAIL (Figure 2E). These results indicated that metformin could induce autophagy in TRAIL-resistant human lung adenocarcinoma A549 cells.

### Metformin enhances TRAIL-induced tumor cell death is blocked by autophagy inhibitor

**Figure 3:**
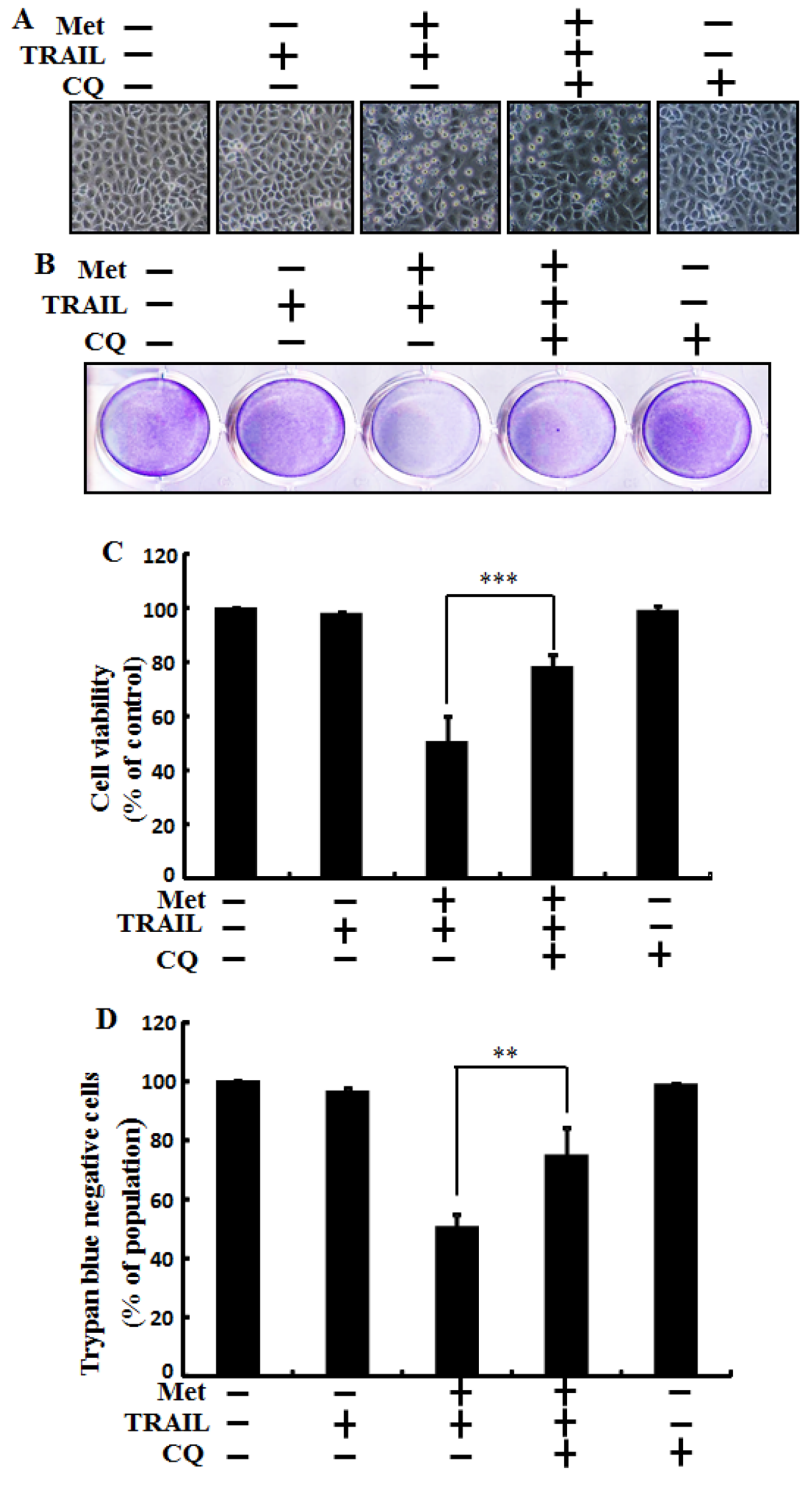
Metformin enhances TRAIL-induced tumor cell death is blocked by autophagy inhibitor A549 adenocarcinoma cells were also pretreated with chloroquine for 1 h followed by treatment with metformin (4 mM) for 12 h. After that, cells were treated with 200 ng/ml of TRAIL protein for an additional 2 h. **A.** Cell morphology photographed under light microscope (×100); **B.** Cell viability measured by crystal violet assay; **C.** Bar graph showing average density of crystal violet dye; **D.** Treated cells measured by trypan blue dye exclusion assays. ** *p*<0.01, *** *p* < 0.001: significant differences between metformin and treatment group. Met: Metformin; TRAIL: Tumor necrosis factor (TNF)-related apoptosis-inducing ligand; CQ: Chloroquine.

Autophagy inhibitor chloroquine was used to determine the effect of metformin on TRAIL induced tumor cell death in human lung adenocarcinoma A549 cells. A549 cells were pretreated with indicated concentration of metformin for 12 h followed by treatment with TRAIL protein for an additional 2h. Additional cells were also pretreated with chloroquine for 1 h followed by metformin treatment. As shown in Figure 3, treatment with TRAIL or chloroquine alone did not influence cell viability or only slightly influenced the cell viability of A549 cells without morphological changes compared to the control. The combined treatment of TRAIL with metformin significantly enhanced cell death. However, co-treatment of metformin, TRAIL, and chloroquine blocked cell death. Cell morphology results also supported that chloroquine blocked cell death effect compared to treatment with metformin and TRAIL (Figure 3A). The co-treatment of metformin, TRAIL, and chloroquine significantly increased cell viability of lung adenocarcinoma A549 cells with decreased cell death (Figure 3B, 3C, and 3D). These results indicated thatautophagy inhibitor chloroquine could promote metformin mediated tumor cell survival induced by TRAIL.

### Autophagy inhibitor blocks TRAIL mediated tumor cell death by metformin via activating autophagy flux

**Figure 4:**
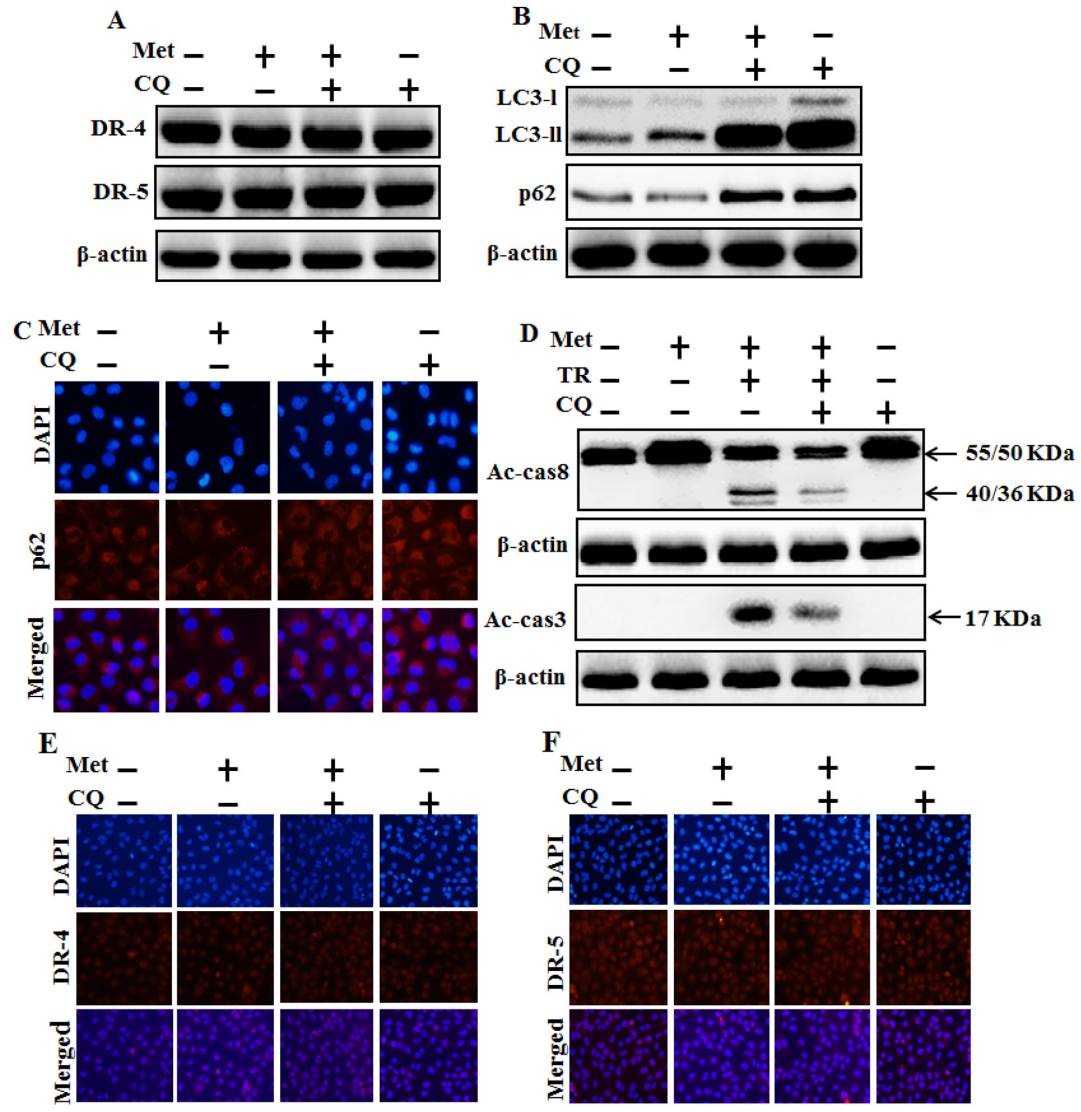
Autophagy inhibitor blocks TRAIL mediated tumor cell death by metformin via regulating autophagy flux A549 adenocarcinoma cells were pretreated with chloroquine for 1h followed by treatment with metformin (4 mM) for 12 h. Cells were harvested and analyzed by Western blotting. **A.** and **B.** Expression levels of DR-4, DR-5, LC3-II, and p62; **C, E.** and **F.** Representative immunocytochemistry of A549 cells after treatment with metformin (12 h) and chloroquine to determine p62 protein levels and surface expression of DR-4 and DR-5 ; **D.** Ac-cas3 and Ac-cas8 expression levels based on western blot analysis. A549 adenocarcinoma cells were pretreated with chloroquine for 1 h followed by treatment with metformin (4 mM) for 12 h. After that, A549 cells were treated with 200 ng/ml TRAIL protein for an additional 1h. β-actin was used as loading control. Met: Metformin; Ac-cas3: Activated caspase 3; Ac-cas8: Activated caspase 8; TRAIL: Tumor necrosis factor (TNF)-related apoptosis-inducing ligand; CQ: Chloroquine.

We investigated the effect of metformin-mediated TRAIL-induced apoptotic pathway by activating autophagy with chloroquine, A549 cells were pretreated with concentration (previously described) of metformin for 12 h and followed by treatment with TRAIL protein for an additional 1h. Additional cells were also pretreated with chloroquine for 1 h followed by metformin treatment. Whole cell lysates were subjected to Western blotting to determine changes in DR4, DR5, LC3-II, P62, Ac-cas3 and Ac-cas8 expression levels. The expression levels of TRAIL receptors such as DR4 and DR5 were not changed by the treatment with metformin or chloroquine alone or by treatment with both metformin and chloroquine in A549 cells (Figure 4A). Autophagy induction was further accepted by the observation of autophagic flux using chloroquine. Chloroquine caused marked accumulation of membrane-bound LC3-II forms, with decreasing P62 (Figure 4B). Immunocytochemistry (ICC) results also revealed that metformin treatment decreased the protein level of p62 compared to chloroquine treatment or combined treatment of metformin and chloroquine (Figure 4C). The combined treatment of TRAIL and metformin enhanced the expression levels of Ac-cas3 and Ac-cas8. However, co-treatment of metformin, TRAIL, and chloroquine blocked the increase in expression level of intracellular apoptosis indicators Ac-cas3 and Ac-cas8 (Figure 4D). Immunocytochemistry (ICC) results revealed that surface expression of DR4 and DR5 were not changed by the treatment with metformin or chloroquine alone or by treatment with both metformin and chloroquine in A549 cells (Figure 4E and 4F). These results indicated that metformin-mediated enhancement of TRAIL-induced apoptotic pathway could be blocked by chloroquine through activation of autophagy flux.

### Metformin enhances TRAIL-induced tumor cell death is blocked by genetic autophagy inhibitor

**Figure 5:**
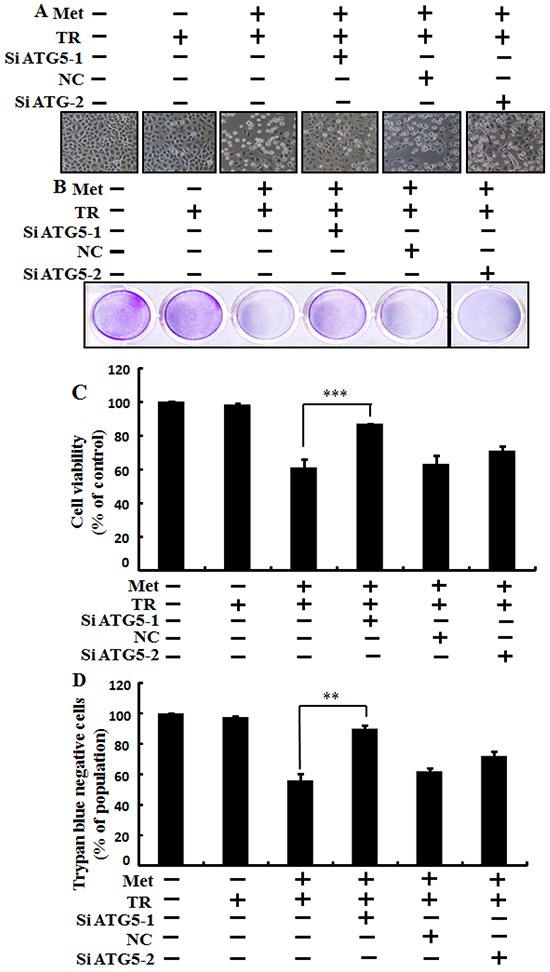
Metformin enhances TRAIL-induced tumor cell death is blocked by genetic autophagy inhibitor A549 adenocarcinoma cells were pretreated with ATG5 siRNA-1and ATG5 siRNA-2 or negative control NC siRNA for 24 h followed by treatment with 4 mM metformin for 12 h with or without 200 ng/ml of TRAIL protein for an additional 2 h. **A.** Cell morphology photographed under light microscope (×100); **B.** Cell viability measured by crystal violet assay; **C.** Bar graph showing average density of crystal violet dye; D. Treated cells measured by trypan blue dye exclusion assays. ** *p*<0.01, *** *p* < 0.001: significant differences between metformin and each treatment group. Met: Metformin; TRAIL: Tumor necrosis factor (TNF)-related apoptosis-inducing ligand; siATG5: ATG5 small interfering RNA; NC: Negative control.

We used Genetic autophagy inhibition ATG5siRNA-1, ATG5siRNA-2 to investigate the effect of metformin on TRAIL induced tumor cell death in human lung adenocarcinoma A549 cells. A549 adenocarcinoma cells were pretreated with ATG5siRNA-1, ATG5siRNA-2 or negative control siRNA (NC) for 24 hour then treated 4mM metformin for 12h with or without 200 ng/ml of the TRAIL protein for an additional 2 h. As shown in Figure 5, treatment with TRAIL alone failed to influence cell viability or only slightly influenced the cell viability of A549 cells without morphological changes compared to the control. Combined treatment with TRAIL and metformin significantly enhanced cell death. However, co-treatment of metformin, TRAIL, and ATG5siRNA-1 blocked cell death. Cell morphology results also revealed that ATG5siRNA-1 blocked cell death compared to treatment with metformin, TRAIL, and control NC siRNA (Figure 5A). Co-treatment with metformin, TRAIL, and ATG5siRNA-1 significantly increased the cell viability of lung adenocarcinoma A549 cells with decreased cell death (Figure 5B, 5C and 5D). These results indicated that geneticautophagy inhibitor ATG5siRNA-1 could promote metformin mediated tumor cell survival induced by TRAIL.

### Genetic autophagy inhibitor blocks TRAIL-induced tumor cell death by metformin via activating autophagy flux

**Figure 6:**
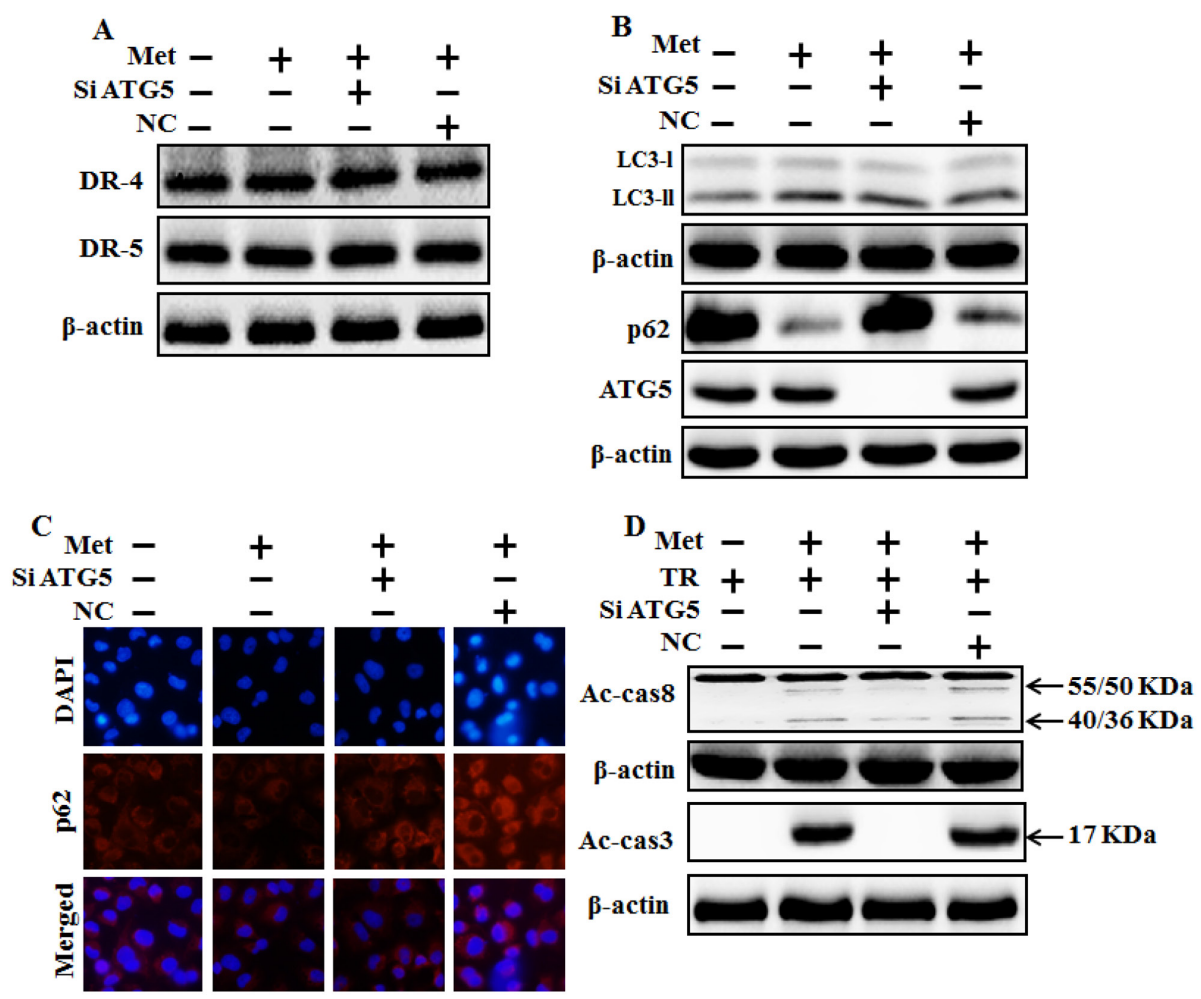
Genetic autophagy inhibitor blocks TRAIL-induced tumor cell death by metformin via regulating autophagy flux A549 adenocarcinoma cells were pretreated ATG5 siRNA or negative control (NC) siRNA for 24 h followed by treatment with 4 mM metformin for 12 h. Cells were harvested and subjected to western blot analysis. **A.** and **B.** Protein levels of DR-4, DR-5, LC3-II, p62 and ATG5; **C.** Protein level of p62 based on immunocytochemistry in A549 cells after treatment with ATG5 siRNA or NC siRNA and metformin for 12h; **D.** Protein levels of Ac-cas3 and Ac-cas8 based on western blot analysis. A549 adenocarcinoma cells were pretreated with ATG5 siRNA or NC siRNA for 24 h followed by treatment with metformin (4mM) for 12 h with or without 200 ng/ml TRAIL for an additional 1h. β-actin was used as loading control. Met: Metformin; Ac-cas3: Activated caspase 3; Ac-cas8: Activated caspase 8; TRAIL: Tumor necrosis factor (TNF)-related apoptosis-inducing ligand; siATG5: ATG5 small interfering RNA; NC: Negative control.

We investigated the effect of metformin-mediated TRAIL-induced apoptotic pathway by activating autophagy withGenetic autophagy inhibition ATG5siRNA. A549 adenocarcinoma cells were pretreated with ATG5siRNA or negative control siRNA (NC) for 24 hour then treated 4mM metformin for 12h with or without 200 ng/ml of the TRAIL protein for an additional 1 h. Whole cell lysates were subjected to Western blotting to determine changes in DR4, DR5, LC3-II, P62, Ac-cas3 and Ac-cas8 expression levels. The expression levels of TRAIL receptors such as DR4 and DR5 were not changed after the treatment with metformin alone or in combination with ATG5siRNA or NC siRNA in A549 cells (Figure 6A). To address the role of autophagy, cells were transfected with siRNA directed against autophagy protein 5 (Atg5) to block autophagic vesicle formation, and silencing of ATG5 was confirmed and knockdown of ATG5 markedly suppressed the metformin-induced LC3-II level (Figure 6B). Immunocytochemistry (ICC) results also supported this protein level of p62 in A549 cells (Figure 6C). Combined treatment of TRAIL, NC siRNA, and metformin enhanced the protein levels of Ac-cas3 and Ac-cas8. However, co-treatment of metformin, TRAIL, and ATG5 siRNA blocked the increase of Ac-cas3 and Ac-cas8 expression levels (Figure 6D). These results indicated that metformin-mediated enhancement of TRAIL-induced apoptotic pathway could be blocked by genetic autophagy inhibitor through activation of autophagy flux.

### Metformin enhances TRAIL-induced apoptosis through downregulation of c-FLIP

**Figure 7:**
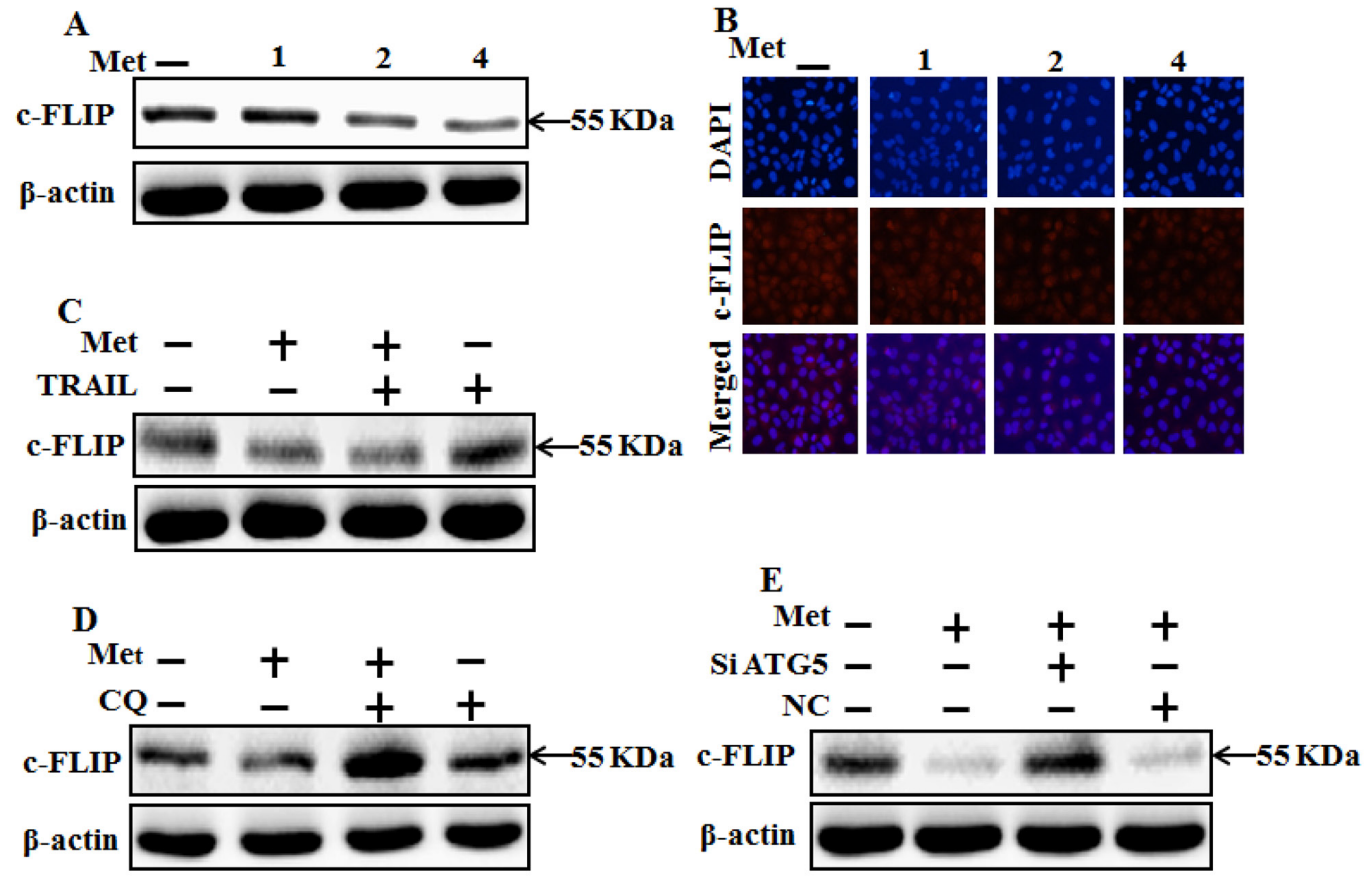
Metformin enhances TRAIL-induced apoptosis through downregulation of c-FLIP A549 cells were pretreated with metformin at different concentrations (0, 1, 2, and 4 mM) for 12 h followed by treatment with 200 ng/ml of TRAIL protein for an additional 1 h. **A, B.** and **C.** Cells were harvested and analyzed by Western blotting and immunocytochemistry to determine the expression levels of c-FLIP; **D.** c-FLIP expression levels based on western blot analysis. A549 adenocarcinoma cells were pretreated with chloroquine for 1 h followed by treatment with metformin (4 mM) for 12 h; **E.** Protein levels of c-FLIP based on western blot analysis. A549 adenocarcinoma cells were pretreated with ATG5 siRNA or NC siRNA for 24 h followed by treatment with metformin (4mM) for 12 h with or without 200 ng/ml TRAIL for an additional 1h. β-actin was used as loading control. Met: Metformin; c-FLIP :cellular FLICE–like inhibitory protein; CQ: Chloroquine; TRAIL: Tumor necrosis factor (TNF)-related apoptosis-inducing ligand; siATG5: ATG5 small interfering RNA; NC: Negative control.

To investigate the mechanism by which metformin sensitizes TRAIL-induced apoptosis, we examined the modulatory effect of metformin on c-FLIP, which are the key components of the TRAIL signaling pathway. However, western blot and immunocytochemistry data showed the expression of c-FLIP was decreased by metformin in a dose dependent manner (Figure 7A and 7B). The combination of metformin and TRAIL reduced c-FLIP levels in A549 cells (Figure 7C). Autophagy induction was further accepted by the observation of autophagic flux using chloroquine. c-FLIP protein increased by chloroquine in co-treatment of metformin (Figure 7D). Furthermore, Combination of ATG5siRNA and higher concentrations of metformin increased c-FLIP compared to metformin treatment alone (Figure 7E). These results indicated that c-FLIP is the key determinant in mediating TRAIL resistance in A549 cells and metformin downregulates c-FLIP and enhances TRAIL-induced apoptosis via activating autophagy flux.

## DISCUSSION

The purpose of this study was to determine the effect of metformin with or without TRAIL on human lung adenocarcinoma A549 cells. Our results suggested that metformin could enhance TRAIL-induced cancer cell death via activating autophagy flux in human lung adenocarcinoma A549 cells. Combination chemotherapy is an impressive strategy to increase the efficacy of anticancer drugs while diminishing their side effects and alleviating drug resistance. TRAIL is regarded as one of the most interesting and promising discovered anticancer agents because it can induce apoptosis of specific cancer cells [3, 32–36], leading to the destruction of cancer cells without showing any toxicity to normal cells. Despite favorable preclinical *in vitro* and *in vivo* results [37–39] of TRAIL as a single agent in first clinical trials, applying TRAIL has been disconcerting since only moderate overall anti-tumor activity has been achieved in patients with advanced malignancies [40]. Downregulation of c-FLIP by chemicals or siRNA has been exposed to sensitize different types of cancer cells to TRAIL; thus, c-FLIP has been contemplated the key negative governor of TRAIL/death receptor-mediated apoptosis [41]. Metformin, a biguanide, is an orally potent synthetic antidiabetic drug used by approximately 120 million people worldwide [42, 43]. Metformin can inhibit the growth of prostate cancer [12] and breast cancer [44] in mouse xenograft model. Apoptosis plays an important role in the regulation of carcinogenesis [45] where apoptotic pathways are interesting targets for the development of new anti-cancer drugs [46]. Autophagy is an evolutionarily conserved mechanism by which cytoplasmic materials including damaged proteins and organelles can be segregated for lysosome-dependent degradation [47, 48]. Autophagy can play a role in cancer either through pro-survival or pro-death mechanism [49–51]. The connection between apoptosis and autophagy is controversial, complex, and poorly understood. In some cellular context, autophagy is mandatory for apoptosis. In other cellular systems, autophagy delays or inhibits apoptosis as a stress adaptation. In some cell settings, the two processes occur individually or independently [52–54].

Most primary cancer cell lines and a range of patient-created tumor cell lines are conclusively resistant to the apoptotic effect of TRAIL [55]. Jin *et al* (8) have demonstrated that lung adenocarcinoma A549 cells are resistant to TRAIL. In this study, we also demonstrated that a single treatment of metformin or TRAIL failed to induce cell death or only slightly increased cell death of human lung adenocarcinoma A549 cells. We found that co-treatment of metformin with TRAIL significantly induced cell death of human lung adenocarcinoma A549 cells that were resistant to either agent alone (Figure 1). This indicates that antidiabetic drug metformin can be used to sensitize TRAIL-mediated cell death in TRAIL-resistant human lung adenocarcinoma A549 cells. Some reports have shown that metformin treatment can induce apoptosis in some types of cancer [56–58] and autophagy of colon, melanoma, and lymphoma cancers [56, 59, 60]. However, our western blot and immunocytochemistry results demonstrated that treatment with various concentrations of metformin alone resulted in increased LC3-II accumulation but decreased p62 levels in human lung adenocarcinoma A549 cells in a dose-dependent manner, while co-treatment of metformin with TRAIL enhanced the protein levels of Ac-cas3 and Ac-cas8 compared to treatment with metformin or TRAIL alone (Figure 2). It has been reported that direct anticancer effect of metformin can activate AMPK, a critical cellular energy sensor and suppression of mTOR signaling, leading to the inhibition of protein synthesis and cell proliferation [16]. Our results revealed that combined treatment of metformin and TRAIL enhanced cell death. Expression of c-FLIP was decreased by metformin in a dose dependent manner and combination of metformin and TRAIL reduced c-FLIP levels in A549 cells. However, pharmacological inhibition of autophagy by co-treatment of chloroquine, metformin, and TRAIL promoted the survival of human lung adenocarcinoma A549 cells. In addition, genetic autophagy inhibitor ATG5 siRNA blocked metformin mediated tumor cell death of A549 cells induced by TRAIL. These results suggest that metformin downregulation of c-FLIP and could enhance TRAIL-induced cancer cell death via regulating autophagy flux in human lung adenocarcinoma A549 cells.

In conclusion, metformin downregulation of c-FLIP and could enhance TRAIL-induced apoptosis via activating autophagy flux. Combined treatment of TRAIL with metformin might be an adequate therapeutic strategy to safely treat some TRAIL-resistant human cancers including lung adenocarcinoma cells.

## MATERIALS AND METHODS

### Cell culture

Cancer cells originating from lung (A549, Calu-3 and HCC-15) tumors were obtained from the American Type Culture Collection (Global Bioresource Center, Manassas, VA, USA). Cells were cultured in RPMI-1640 and MEM (Gibco BRL, Grand Island, NY, USA) medium supplemented with 10% (v/v) fetal bovine serum and antibiotics (100 μg/ml penicillin-streptomycin). Cells were cultured at 37°C supplied with 5% CO_2_.

### Reagents

Recombinant metformin and chloroquine (20 μM) were purchased from Sigma-Aldrich (St. Louis, MO, USA). TRAIL (200 ng/ml) was purchased from Abfrontier (Geumcheon-gu, Seoul, South Korea).

### Cell viability test

A549, Calu-3 and HCC-15 cells were seeded at 1.0 × 10^4^ cells onto 12-well plates and incubated at 37°C for 24 h. These A549, Calu-3 and HCC-15 cells were pretreated with metformin at different concentrations (0, 1, 2, and 4 mM). After 12-h pretreatment with metformin, recombinant TRAIL protein (200 ng/ml) was added and co-incubated for 2 h. Additional cells pretreated with chloroquine (20 μM) for 1 h were also treated with metformin. Cell morphology was assessed under microscopy (inverted Microscope, Nikon, Japan). Cell viability was determined using the crystal violet staining method. Briefly, cells were stained with a staining solution (0.5% crystal violet in 30% ethanol and 3% formaldehyde) for 10 min at room temperature, washed four times with PBS, and dried. Cells were then lysed with 1% SDS solution. The absorbance value was then measured at wavelength of 550 nm using a plate reader. Cell viability was expressed as relative dye intensity compared to that of the control.

### Trypan blue exclusion assay

The number of viable cells was determined with trypan blue dye exclusion (Sigma-Aldrich) using a hemocytometer. The result was expressed as a percentage of viable cells compared to that of vehicle-treated controls.

### Western blot assay

Cells were harvested, washed in cold PBS, resuspended in lysis buffer [25 mM HEPES (pH 7.4), 100 mM EDTA, 5 mM MgCl_2_, 0.1 mM DTT, and a protease inhibitor mixture], and sonicated to prepare A549 cell lysates. Proteins (35 μg) were separated in 10–15% SDS gels and transferred to nitrocellulose membranes. After incubation with 1:1000 primary antibody in dilution buffer (1% milk with PBS-Tween) and secondary antibody, Membranes were developed with enhanced chemiluminescence. The following antibodies were used for immunoblotting: LC3 (Novus Biologicals, Littleton, CO, USA), anti-P62 (Millipore Corp., Milford, MA, USA), cleaved caspase-3 (Cell Signaling Technology, Danvers, MA, USA), cleaved caspase-8 (BD pharmingen, USA), c-FLIP (Enzo life sciences, USA), DR-4, DR-5, and β-actin Sigma-Aldrich (St. Louis, MO, USA). Images were obtained using a Fusion-FX7 imaging system (Vilber Lourmat, Marne-la-Vallée, France).

### Immunocytochemistry

A549 cells cultured on glass coverslips were treated with metformin, chloroquine, and/or TRAIL, washed with PBS, and fixed with 3-4% paraformaldehyde in PBS at room temperature (RT) for 15 min. Cells were then washed twice with ice cold PBS and incubated at RT for 10 min in PBS containing 0.25% Trion X-100. After the incubation, cells were washed three times with PBS (5 minutes each wash). After blocking with 1% BSA in PBST for 30 minutes, cells were incubated with primary antibody (anti-p62, anti c-FLIP diluted with 1% BSA in PBST) in a humidified chamber at room temperature for 1 hour or at 4°C overnight. After the incubation, solution was decanted and cells were washed three times with PBS (5 minutes each wash). Cells were then incubated with secondary antibody (diluted with 1% BSA in PBST) for 1 hour at room temperature in the dark. After the incubation, the solution was decanted and cells were washed with PBS three times (5 minutes per wash). Cells were then incubated with DAPI for 1 minute followed by PBS rinse. Finally, cells were mounted with fluorescent mounting medium and visualized using a fluorescence microscope.

### TEM (transmission electron microscopy) analysis

TEM samples were analyzed by Transmission Electron Microscope (JEM-2010, JEOL) installed in the Center for University-Wide Research Facilities (CURF) at Chonbuk National University. After fixation of A549 cell samples in 2.5% glutaraldehyde (TED PELLA, USA) in PBS (pH, 7.2), specimens were post fixed in 1% osmium tetroxide (Heraeus, South Africa), dehydrated in graded ethanol and propylene oxide (Acros Organics, USA), and then embedded in Epoxy resin(Embed812. NMA; Nadic methyl anhydride. DDSA; Dodenyl Succinic Anhidride. DMP-30., USA) as used previously. Serial ultrathin sections were cut on LKB-III ultratome (LEICA, Germany). Ultrathin sections were stained with uranyl acetate (TED PELLA, USA) and lead citrate (TED PELLA, USA) and examined with the aid of a Hitachi H7600 electron microscope (Hitachi, Japan) at an accelerating voltage of 100 kV.

### RNA interference

A549 cells were transfected with ATG5 small interfering RNA (siRNA-1; oligo ID HSS114103 and siRNA-2; oligo ID HSS190366; Invitrogen, Carlsbad, CA, USA) using Lipofectamine 2000 transfection reagent (Invitrogen) according to manufacturer's instructions. At 36-h post transfection, the knockdown efficiency at protein level was determined by immunoblotting and cell viability test. Scrambled siRNA (Invitrogen) was used as negative control.

### Statistical analysis

Unpaired t-test or Welch's correction was used for comparison between two groups. For multiple comparisons, one-way analysis of variance (ANOVA) followed by Tukey-Kramer test was used. All statistical analysis was performed using GraphPad Prism software. Statistical significance was considered when *p* value was less than 0.05 (*), 0.01 (**), or 0.001 (***).
